# Mass spectrometry detection of inhaled drug in distal fibrotic lung

**DOI:** 10.1186/s12931-022-02026-5

**Published:** 2022-05-11

**Authors:** Theresia A. Mikolasch, Eunice Oballa, Mitra Vahdati-Bolouri, Emily Jarvis, Yi Cui, Anthony Cahn, Rebecca L. Terry, Jagdeep Sahota, Ricky Thakrar, Peter Marshall, Joanna C. Porter

**Affiliations:** 1 Centre for Inflammation and Tissue Repair, UCL Respiratory, University College London, London, UK; 2 Discovery Medicine, Clinical Pharmacology and Experimental Medicine, GSK Research and Development, Stevenage, UK; 3 Early Development Leader, GSK Research, Stevenage, UK; 4 Development Biostatistics, GSK Development, Stevenage, UK; 5 Safety and Medical Governance, Pharma Safety, GSK Development, Stevenage, UK; 6 Pathology, In Vitro/In Vivo Translation, GSK Research, Stevenage, UK; 7 University College London Hospitals NHS Foundation Trust, London, UK; 8 Bioimaging, In Vitro/In Vivo Translation, GSK Research, Stevenage, UK

**Keywords:** MALDI-MS imaging, Transbronchial cryobiopsy, Drug distribution, Interstitial fibrosis

## Abstract

**Background:**

Currently the only available therapies for fibrotic Interstitial Lung Disease are administered systemically, often causing significant side effects. Inhaled therapy could avoid these but to date there is no evidence that drug can be effectively delivered to distal, fibrosed lung. We set out to combine mass spectrometry and histopathology with rapid sample acquisition using transbronchial cryobiopsy to determine whether an inhaled drug can be delivered to fibrotic, distal lung parenchyma in participants with Interstitial Lung Disease.

**Methods:**

Patients with radiologically and multidisciplinary team confirmed fibrotic Interstitial Lung Disease were eligible for this study. Transbronchial cryobiopsies and endobronchial biopsies were taken from five participants, with Interstitial Lung Disease, within 70 min of administration of a single dose of nebulised ipratropium bromide. Thin tissue cryosections were analysed by Matrix Assisted Laser Desorption/Ionization-Mass Spectrometry imaging and correlated with histopathology. The remainder of the cryobiopsies were homogenised and analysed by Liquid Chromatography—tandem Mass Spectrometry.

**Results:**

Drug was detected in proximal and distal lung samples from all participants. Fibrotic regions were identified in research samples of four of the five participants. Matrix Assisted Laser Desorption/Ionization-Mass Spectrometry imaging showed co-location of ipratropium with fibrotic regions in samples from three participants.

**Conclusions:**

In this proof of concept study, using mass spectrometry, we demonstrate for the first-time that an inhaled drug can deposit in distal fibrotic lung parenchyma in patients with Interstitial Lung Disease. This suggests that drugs to treat pulmonary fibrosis could potentially be administered by the inhaled route.

*Trial registration* A prospective clinical study approved by London Camden and Kings Cross Research Ethics Committee and registered on clinicaltrials.gov (NCT03136120)

**Supplementary Information:**

The online version contains supplementary material available at 10.1186/s12931-022-02026-5.

## Background

The interstitial lung diseases (ILDs) are a group of over 200 lung disorders that are characterised by interstitial fibrosis, and lead to declining lung function, respiratory failure and ultimately death. The most severe fibrotic (f)ILD is Idiopathic Pulmonary Fibrosis (IPF).

Two oral drugs, pirfenidone and nintedanib, are licensed for the treatment of IPF and have now been shown to have benefits in other fILDs [1] but both have limiting adverse effects. Inhaled therapy for ILD offers the advantage of drug delivery direct to the lung, thereby minimising systemic exposure and associated side effects. However, lung deposition, absorption and local therapeutic response may be altered in the fibrotic lung [2].

Assessment of lung drug levels using bronchoscopic lavage has become a critical component of inhaled drug development but lacks spatial information of the site or region of deposition. The advent of transbronchial cryobiopsy (TBC) to sample the lung parenchyma for diagnosis of ILD allows important histological information of inhaled drug distribution using a minimally invasive bronchoscopic technique [3]. TBC potentially also allows more rapid lung tissue sampling following drug inhalation, compared to traditional surgical lung biopsies therefore shortening the time during which the inhaled drug can be cleared from the lung before analysis. Furthermore, participants are not subject to mechanical ventilation which could theoretically alter inhaled drug distribution in surgical participants.

Liquid Chromatography—tandem Mass Spectrometry (LC–MS/MS) is traditionally used for the analysis of homogenised tissue samples and therefore any spatial information regarding drug distribution within the tissue is lost. In contrast, Matrix Assisted Laser Desorption Ionisation—Mass Spectrometry (MALDI-MS) imaging allows detection and characterisation of molecules from tissue [4–15] and supports the spatial visualisation of drug distribution in tissue samples. Analysing the same TBC biopsy with a combination of LC–MS/MS, MALDI-MS imaging and histopathology can therefore allow minimally invasive assessment of drug distribution within the diseased fibrotic lung.

Inhaled ipratropium was chosen for this study for several reasons: it is a quaternary ammonium compound and therefore strongly positively charged which facilitates detection with MALDI-MS; during the feasibility stage, different drugs (anti-cholinergics, beta-agonists, steroids, and mast cell stabilisers) were tested for of detection using MALDI and secondary-ion mass spectrometry (SIMS) and only ipratropium bromide had good sensitivity for both; ipratropium has been used extensively with robust safety data; and the high therapeutic index and wide therapeutic window of ipratropium also afforded us the ability to increase drug dosing if required. Fehniger et al. [15] have previously demonstrated inhaled Ipratropium distribution using MALDI-MS imaging in the proximal airways of patients with suspected airway obstruction or tumours. Ipratropium readily ionises and the MS/MS fragmentation pattern produces two major fragment ions (at m/z 166.0 and 123.9, (Additional file 1: Fig. E1).

In this clinical study, having carried out a single pre-clinical enabling study, we combined, for the first time, rapid distal sample acquisition using TBC with the mass spectrometry modalities of LC–MS/MS and MALDI-MS imaging, together with histopathology to demonstrate inhaled drug delivery to fibrotic, distal human lung parenchyma in participants with diagnosed ILDs. Whilst this study was designed as a proof of concept, with a low participant number (n = 5), we are able to present confirmation that inhaled drug therapy is a feasible route of administration for fibrotic ILD, which could avoid the significant systemic side effects of current oral therapy. To our knowledge this is also the first time that TBC has been used in translational research.

## Methods

### Pre-clinical support study in wistar han rats

All animal studies were ethically reviewed and carried out in accordance with UK Animals (Scientific Procedures) Act 1986, European Directive 2010/63/EU and the GSK Policy on the Care, Welfare and Treatment of Laboratory Animals.

A scaled dose of ipratropium bromide, equivalent to the clinical dose, was nebulised to male Wistar rats for 5 min. Terminal lung samples were taken at varying time points up to 65 min post-dose and 5 mm ex-vivo biopsies were embedded into material suitable for MALDI-MS imaging.

For further details of the pre-clinical animal work please see Additional file 1**.**

### Clinical study

We conducted a prospective clinical study approved by London Camden and Kings Cross Research Ethics Committee and registered on clinicaltrials.gov (NCT03136120) at University College London Hospital (UCLH), London, United Kingdom and sponsored by GlaxoSmithKline. Participants over the age of 18 with suspected ILD and requiring TBC for further diagnostic assessment, as determined by the ILD multidisciplinary team, were eligible to participate (see Additional file 1 for full exclusion/inclusion criteria). Most patients had moderately impaired lung function (FVC between 50 and 80 percent of predicted). Patients with severe ILD were excluded due to risks of additional biopsies in this patient group. Seven participants were enrolled between November 2017 and November 2018.

All participants received a single dose of 500 mcg nebulised ipratropium bromide (Ivax Pharmaceuticals, London, UK) 1 h prior to bronchoscopy. TBCs for diagnosis were taken ahead of the additional (one or two) TBC research samples. Up to three endobronchial forceps biopsy samples were also taken as positive controls to confirm drug inhalation by the participant (see Additional file 1 for full procedural and biopsy collection details).

### Liquid chromatography—tandem mass spectrometry (LC–MS/MS) analysis

Following sectioning of biopsy samples for MALDI-MS imaging, the remainder of the biopsy samples were analyzed by LC–MS/MS for confirmation of drug presence. For more details see Additional file 1.

### MALDI MSI analysis

For experimental conditions and more details see Additional file 1.

In the MALDI-MS imaging experiments the ipratropium cation was detected and will be referred to as ipratropium or drug. Predetermined specific mass transitions for ipratropium (m/z 332.2–166.0 and 332.2–123.9) were utilised. Following smoothing and baseline correction, a signal to noise threshold ratio of 3:1 was applied to both fragment ions (166.0 and 123.9) for detection of ipratropium. A spatial resolution of either 30, 100 or 200 µm was utilised and the signal for ipratropium was displayed using a colour coded ion density map.

### Histopathology

Biopsy sections were stained with haematoxylin and eosin (H&E) following standard histological procedures [16]. Images were captured digitally and scanned at either 20 × or 40 × magnification (Aperio Scanscope CS, Leica Microsystems, Milton Keynes, UK).

## Results

### Pre-clinical study in rat

**Fig. 1:**
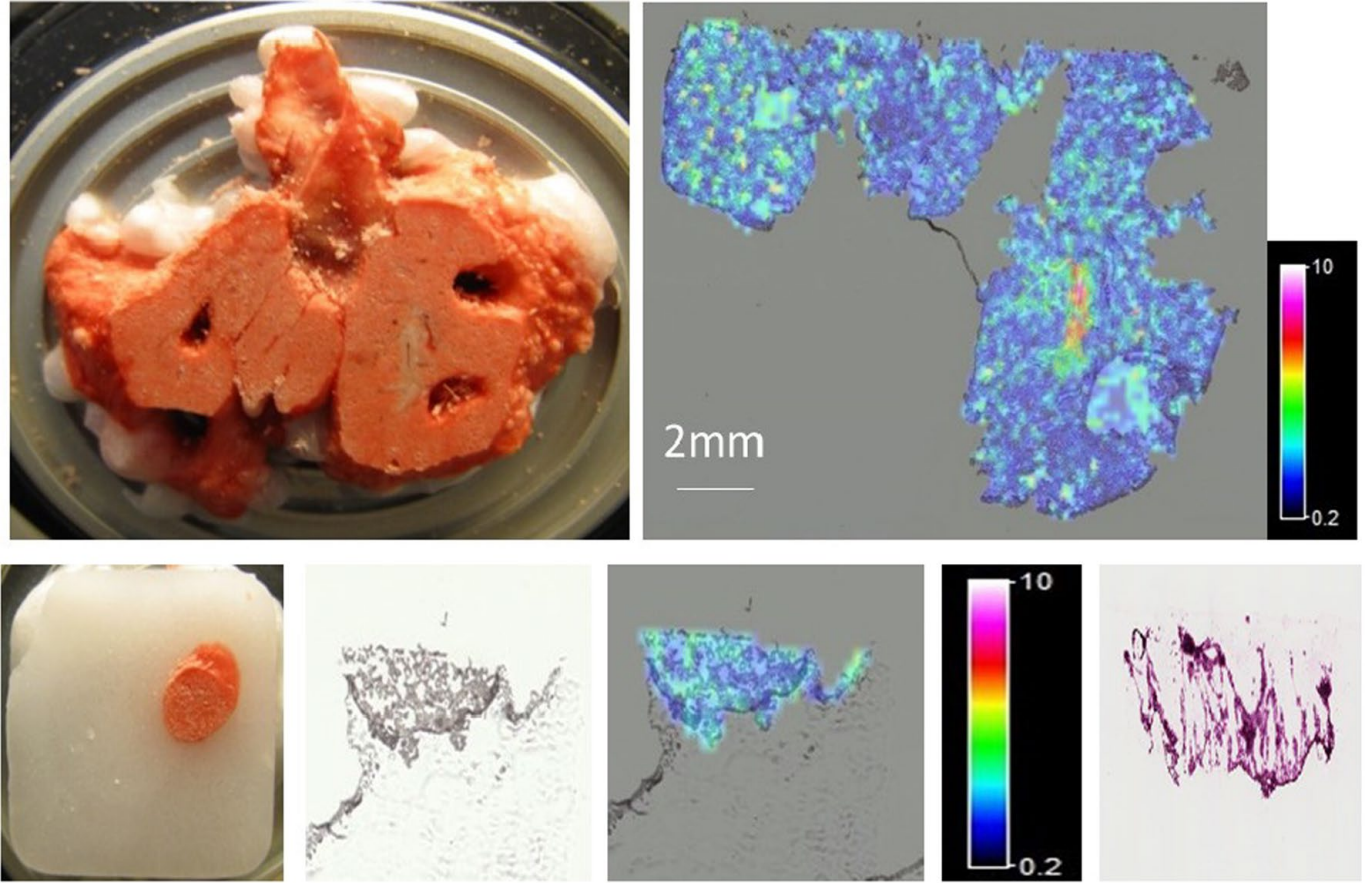
Pre-Clinical Study in rats—MALDI-MS imaging of a 16 µm thick tissue section of rat lung and a 5 mm punched biopsy of rat lung, taken 65 min after a nebulised administration of ipratropium. Top Left—Photo of the region of the tissue from where the section had been cut (after removal of multiple punched biopsies). Top Right—MALDI-MS Image showing the distribution of the *m/z* 166 fragment ion, representative of ipratropium in Rat 7, section 13 (200 µm spatial resolution). The signal intensity for the ipratropium fragment ion at *m/z* 166.0 is represented as a concentration-dependent colour scale—white being highest concentrations. Bottom Left to Right: **a** Photo of 5 mm punched biopsy from rat lung. **b** Optical Image (digitally scanned image of rat lung section). **c** MALDI-MS image, 200 µm spatial resolution (and Signal Intensity Scale bar). **d** Histology image (Consecutive section)

In our pre-clinical study, we demonstrated good MALDI-MS sensitivity for ipratropium with widespread distribution of the drug in the lung using both 30 µm and 200 µm spatial resolution at all timepoints (Fig. 1).

Ipratropium was also measured in rat lung sample sections by LC–MS/MS with the mean amount across samples from all timepoints, up to 65 min post administration, in the low pg/section region. This pre-clinical study demonstrated that the sample handling methods used and the MALDI-MS imaging detection limits for ipratropium in lung tissue were suitable.

### Clinical study

Seven participants were enrolled of whom five completed the trial providing six TBC samples ranging from 4 to 6 mm^2^ in size and fourteen endobronchial biopsy samples ranging from 0.75 to 3 mm^2^ in size. TBC samples were taken within 70 min of the end of ipratropium nebulisation.

**Fig. 2:**
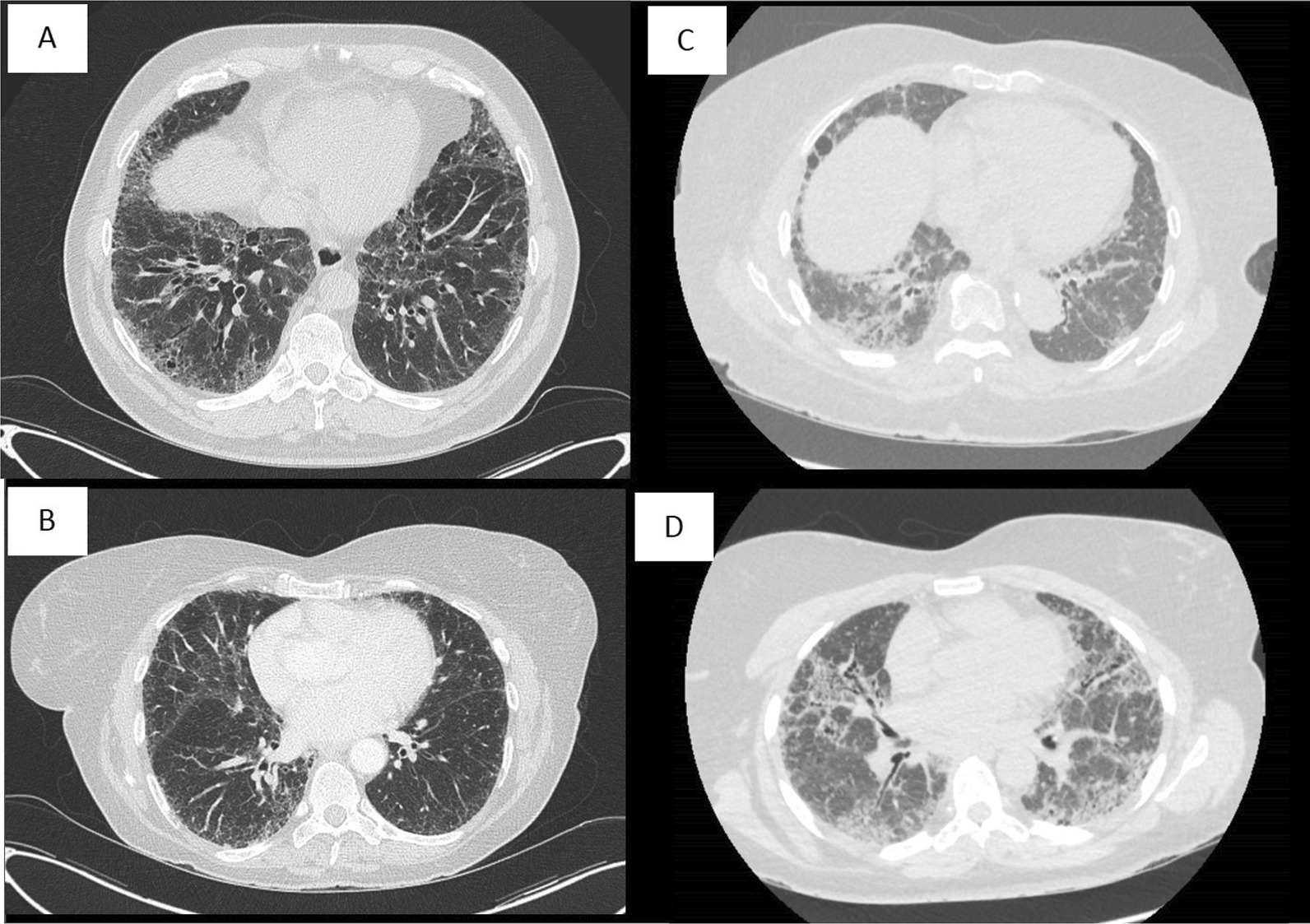
Representative CT scans from 4 patients prior to cryobiopsy from Right Lower Lobe in each case: **A** Patient 001. **B** Patient 003. **C** Patient 004. **D** Patient 006

Participants’ characteristics are summarized in Table 1. Representative CT scans are shown at site of biopsies (Right Lower Lobe in all cases) from 4 of the patients in Fig. 2.

**Table 1:** Summary of participants’ characteristics

	Ipratropium bromide (N* = 7)
Age in Years [Mean (SD)]	62.1 (5.98)
Male [n (%)]	3 (43)
BMI (kg/m^2^) [Mean (SD)]	31.11 (3.191)
Height (cm) [Mean (SD)]	168.71 (16.039)
FVC [Mean (SD)]	2.58 (1.161)
% predicted FVC [Mean (SD)]	73.75 (12.863)
FEV1 [Mean (SD)]	2.12 (0.773)
% predicted FEV1 [Mean (SD)]	77.07 (11.256)

MDT diagnosis of study completers	Study completers (N = 5)
IPF/probable IPF [n (%)]	3 (60)
NSIP/fibrotic NSIP [n (%)]	2 (40)

*BMI* body mass index, *FVC* forced vital capacity, *FEV1* forced expiratory volume at 1 s, *MDT* multidisciplinary team, *IPF* idiopathic pulmonary fibrosis, *NSIP* non-specific interstitial pneumonia

Note 1: ^*^One participant had to be re-enrolled

Note 2: As this was a non-quantitative, proof of concept study the impact of the participant’s characteristics was not designed to be taken into consideration

Two participants were withdrawn at the bronchoscopist’s discretion, prior to research samples being taken, one, due to endobronchial bleeding and the second due to technical difficulties leading to a prolonged procedure.

### Adverse events

The adverse events (AE) reported for the study are presented in Table 2.

**Table 2:** Summary of adverse events

Preferred term	Ipratropium bromide (N = 7)
Any event, n (%)	7 (100)
Procedural haemorrhage^1^	5 (71)
Procedural pneumothorax	2 (29)
Cough	1 (14)
Dry throat	1 (14)
Constipation	1 (14)
Malaise	1 (14)
Musculoskeletal chest pain	1 (14)

^1^Bleeding (procedural haemorrhage) is an expected adverse event associated with biopsy procedures. In this study, for one participant the procedure was stopped before biopsies due to bleeding. For all other participants, bleeding was mild and managed as per UCLH routine procedure

Three participants in this study had serious AEs reported namely pneumothorax (n = 2) and malaise (n = 1).

### Drug detection by LC–MS/MS

LC–MS/MS was carried out. Ipratropium was detected in all six TBC samples tested (Table 3). No quantification data is available due to insufficient TBC or endobronchial control material being available to prepare calibration standards. In addition, an identical liquid volume was used to produce homogenate for each sample irrespective of their differing weights (to aid detection).

**Table 3:** Summary of ipratropium detection by LC–MS/MS

Biopsy sample ID	Biopsy type	Ipratropium detected (±)	Sample weight (mg)
3A	TBC (Distal)	+	3.4
4A	TBC (Distal)	+	20.4
5A	TBC (Distal)	+	23.2
6A	TBC (Distal)	+	0.1
6B	TBC (Distal)	+	7.8
8A	TBC (Distal)	+	97.8
3B	Endobronchial (Proximal)	+	< 0.1
3C	Endobronchial (Proximal)	+	2.6
4B	Endobronchial (Proximal)	+	< 0.1
4C	Endobronchial (Proximal)	+	0.1
4D	Endobronchial (Proximal)	+	1.9
5B	Endobronchial (Proximal)	+	0.1
5C	Endobronchial (Proximal)	+	< 0.1
5D	Endobronchial (Proximal)	No Sample*	1.2
6C	Endobronchial (Proximal)	+	< 0.1
6D	Endobronchial (Proximal)	+	< 0.1
6E	Endobronchial (Proximal)	+	< 0.1
8B	Endobronchial (Proximal)	+	< 0.1
8C	Endobronchial (Proximal)	+	< 0.1
8D	Endobronchial (Proximal)	+	0.5

*Endobronchial biopsy sample 5D was lost during sample preparation

Drug was detected by LC–MS/MS in thirteen endobronchial biopsy samples tested.

### MALDI-MS imaging results

**Fig. 3:**
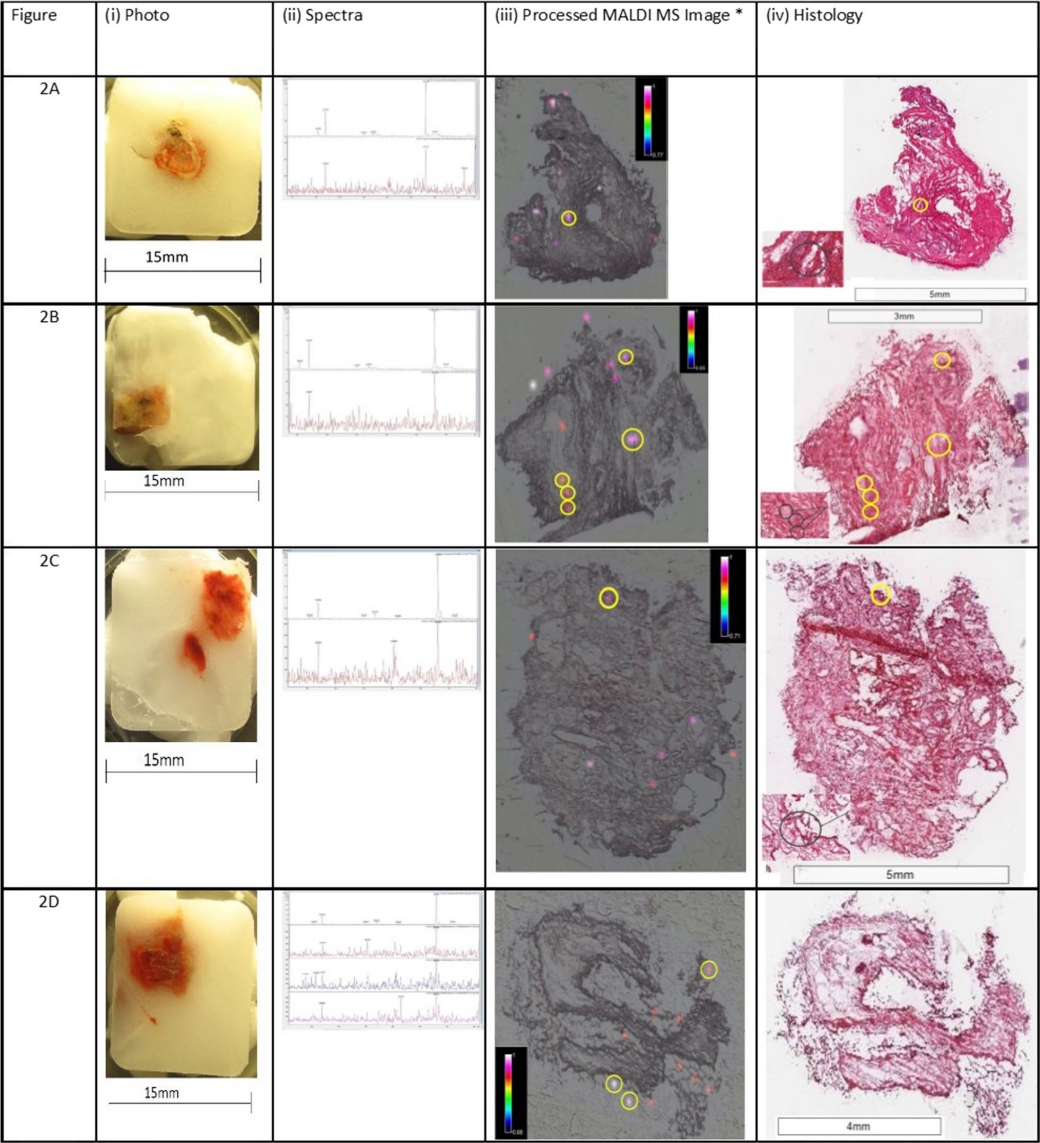
Representative MALDI-MS images, histology images and MS/MS for each participant. (TBC Samples). Each representative figure depicts the MALDI-MS image (100 µm pixel size) for the biopsy sample section and its corresponding histology image, a photograph of the frozen embedded biopsy sample and mass spectra showing both fragment ions (at m/z 123.9 and 166.0), obtained at the site of confirmed ipratropium detection (referred to as ipratropium or drug foci). For clarity, the MALDI-MS images for the detection of ipratropium have been adapted and the drug foci regions circled that are above the signal to noise threshold ratio 3:1 for both fragment ions (at *m/z* 123.9 and 166.0). The approximate location of these foci has been circled on the corresponding histology image

**Fig. 4:**
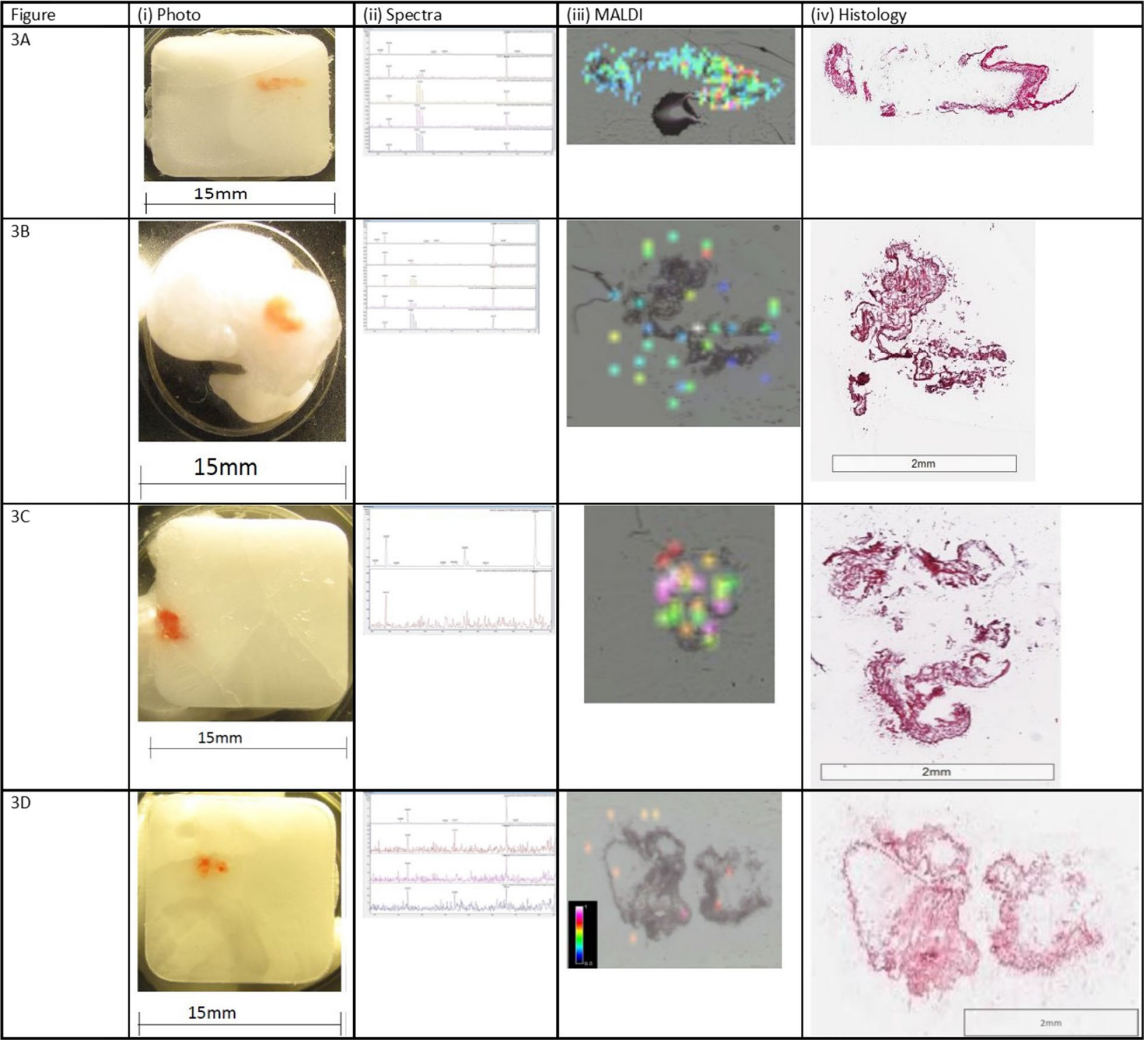
Representative MALDI-MS images, histology images and MS/MS for each participant (endobronchial samples). Each representative figure depicts the MALDI-MS image (100 µm pixel size) for the biopsy sample section and its corresponding histology image, a photograph of the frozen embedded biopsy sample and mass spectra showing both fragment ions (at m/z 123.9 and 166.0), obtained at the site of confirmed ipratropium detection (referred to as ipratropium or drug foci)

Drug was detected in TBC samples from four of the five participants using MALDI-MS imaging. Representative figures for the detection of ipratropium in distal and proximal lung section samples from 4 participants are shown in Figs. 3 and 4 respectively. The circled regions in Fig. 3 (and all regions in Fig. 4) represent the drug foci regions meeting the selection criteria (see Additional file 1) for the positive identification and detection of ipratropium.

### TBC (distal lung) MALDI-MS imaging

Ipratropium was detected in TBC sections as either a single foci or multiple foci using MALDI-MS imaging (Fig. 3). The sample shown in Fig. 3B (iii) contains five ipratropium foci. Three of these foci are adjacent to each other and appear to be co-located with an airway.

**Fig. 5:**
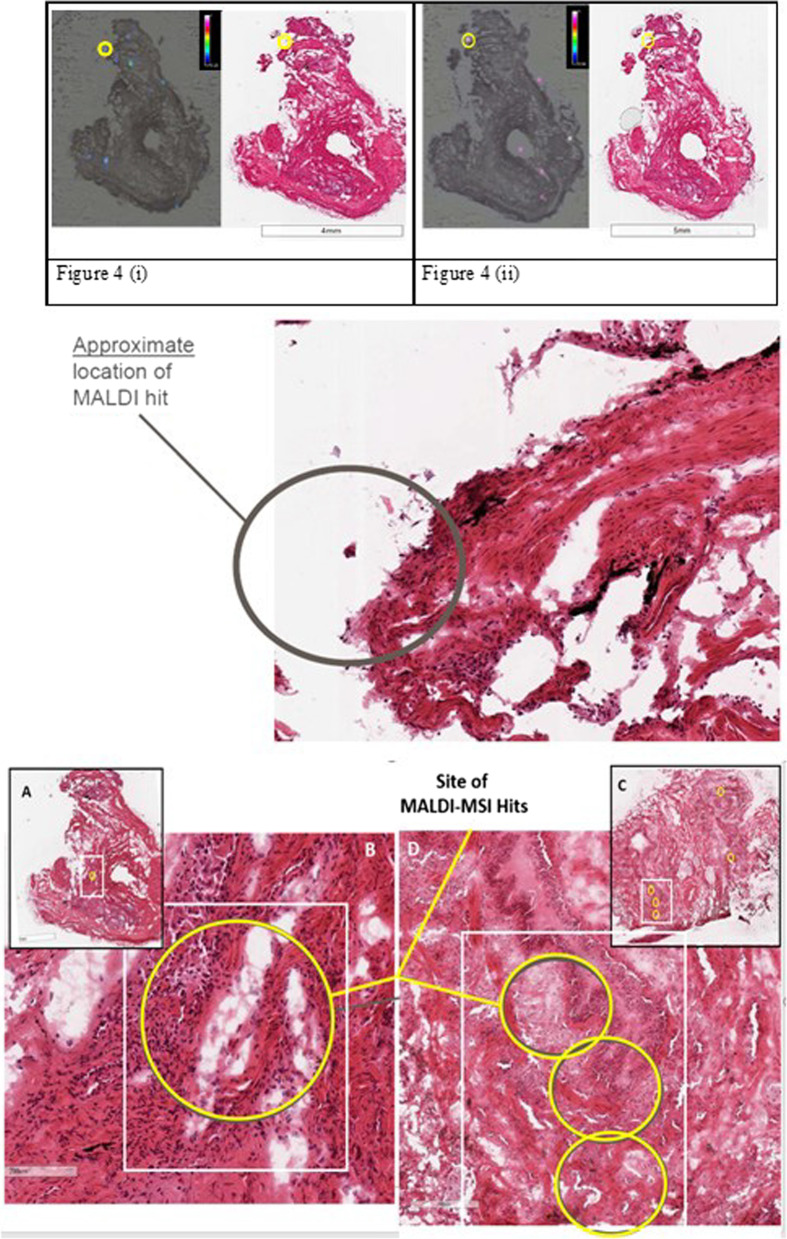
Images showing MALDI-MS imaging hit on consecutive sample sections, 4A32 (i) and 4A33 (ii) and approximate location of MALDI hit (middle). Bottom: **A** Co-location of MALDI-MS imaging and Histology in TBC and Zoomed-in region **B** depict the approximate location of the MALDI-MSI hit present within a fibrotic area of TBC sample 4A31, possibly co-located with a small airway. **C** and zoomed-in region **D** of TBC sample 5A23 illustrate lung architecture consistent with pulmonary fibrosis and the approximate location of the MALDI-MSI hits appear to co-locate with a small airway

Ipratropium loci were observed to localise in the same region in consecutive biopsy sections (Fig. 5i and ii) suggesting an alignment through the z-plane.

### Co-location of MALDI-MS imaging and histology in TBC

Fibrotic regions were identified in biopsies of four of the five participants as indicated by coalescing areas of poorly cellular eosinophilic fibrillar material (interpreted as collagen). Combining the MALDI-MS images and histology demonstrated co-location of ipratropium with fibrotic regions in the TBCs of three of the four participants with fibrosis.

Whilst the number of drug foci within the TBC sections was low, there were examples from three participants, Figs. 3A (iv), B (iv) and C (iv) where the drug foci were shown to co-locate with areas of fibrosis. This indicates that for these three participants, ipratropium bromide could be deposited in regions of the distal lung where fibrosis was also confirmed.

In TBC sections from two participants, drug foci were present within abnormal fibrotic areas (Fig. 5A–D), possibly co-located with small airway, however, low resolution of the image does not allow a full histological interpretation.

One participant (diagnosed with non-specific interstitial pneumonia) did not have abnormal fibrotic areas observed in the research sample, although ipratropium was successfully detected in the TBC sample from their distal lung.

### MALDI-MS imaging results for the endobronchial biopsy (proximal lung)

Endobronchial biopsy samples were taken as a control to confirm drug inhalation by the participant. The levels of ipratropium were expected to be higher in the proximal airways than in the distal lung.

Ipratropium was detected in at least one endobronchial biopsy sample for each of the participants, see Fig. 4 and Table 4. The highest signal intensity and greatest number of drug foci were observed in endobronchial samples Fig. 4A (iii) and C (iii).

**Table 4:** Summary of average drug detection rates per study sample

Biopsy sample ID	Biopsy type	Number of sections analysed	Number of sections with drug observed	% Success	Average % success for endobronchial samples
4A	Transbronchial	25	5	20	
4B	Endobronchial	26	5	19	
4C	Endobronchial	12	8	67	
4D	Endobronchial	8	3	38	34.8
5A	Transbronchial	14	1	7	
5B	Endobronchial	8	6	75	
5C	Endobronchial	8	8	100	
5D	Endobronchial	4	4	100	90.0
6A	Transbronchial	12	1	8	
6B	Transbronchial	12	0	0	
6C	Endobronchial	9	1	11	
6D	Endobronchial	11	4	36	23.5*
6E^†^	Endobronchial	7	0	0^†^	25^†^
8A	Transbronchial	11	4	36	
8B	Endobronchial	7	1	14	
8C	Endobronchial	9	2	22	
8D	Endobronchial	5	3	60	28.6

*Average success rate while incorporating sample 6E is 23.5%

^†^Due to issues we had with generating suitable sections from sample 6E for MALDI-MS imaging we have recalculated the average success for sample 6 excluding the data from biopsy E, updated result is 25%

### Comparison of drug detection in samples from distal (transbronchial) and proximal (endobronchial) lung

The non-statistical, non-quantitative comparison reported here was conducted to demonstrate that more drug was detected in the proximal regions of the lung compared to the distal regions. Inhaled drugs emitted from a device generating polydisperse particle sizes are more likely to deposit higher amounts of drug in the proximal lung and larger airways than the distal lung and alveoli [17].

The signal for the fragment ions of ipratropium were found to be of greater intensities in endobronchial samples relative to the TBC samples (Fig. 4). Table 4 summarises the average detection success rate by MALDI-MS imaging per biopsy sample. The drug foci were also greater in number across the endobronchial biopsy samples compared to the TBC samples. In addition, except for TBC sample 8A, the proportion of sample sections where drug was detected per biopsy was greater for endobronchial biopsy samples (range 23.5–90%) compared to the TBC samples (range 7–36%). For sample 8 (diagnosed with NSIP), the average detection frequencies for the TBC and endobronchial biopsies were similar at 36% and 28.6%, respectively.

## Discussion

Here we report the first ever successful detection and localisation of inhaled drug in the distal lung of histologically confirmed fibrotic lung parenchyma in participants with a clinical diagnosis of fibrotic ILD. This was achieved through the combination of TBC, LC–MS/MS, MALDI-MS imaging and histopathology.

A scaled preclinical study was initially conducted in rats to optimise assay conditions, due to the anticipated challenges with respect to the detection of a single clinical dose of ipratropium in relatively small lung biopsy samples. The preclinical study allowed sample handling methods and detection limits of ipratropium in rat lung samples (similar in size to human cryobiopsy samples) to be assessed by LC–MS/MS and MALDI-MS imaging. Widespread and even distribution of ipratropium was observed, see Additional file 1**,** in both rat lung sections and equivalent sized biopsies to those expected from TBC.

A total of seven participants were dosed with ipratropium bromide, with five participants providing both TBC and endobronchial samples. LC–MS/MS analysis demonstrated the presence of drug in all participants’ TBCs, suggesting that ipratropium was able to deposit in the distal lung, the area that is most affected in IPF.

Drug aerosol particle size by medical nebuliser is polydisperse, therefore containing a mix of different particle sizes. Particle size was not measured as we did not perform any drug delivery quantification. According to product literature the combination of a Porta Neb compressor (Phillips Respironics, Amsterdam, Netherlands) running at 6 L/min with a SideStream aerosolising chamber (Respironics, Tangmere, UK) achieves a mass median diameter of < 5 µm in 80% of droplets generated. Salbutamol nebulised using the same compressor/nebuliser configuration gave a mean mass median aerodynamic diameter (MMAD) of 2.2 µm (SD 0.4) and a mean geometric standard deviation 3.45 µm (SD 1.1) [18]. Aerosol droplet size influences the location of particle deposition and alveolar deposition peaks at about 1.5 µm [19]. It was therefore reasonable to assume that the compressor/nebuliser configuration would create aerosol droplets of sufficiently small size to reach the target tissue. Due to insufficient TBC or endobronchial control material being available to prepare calibration standards no drug quantification measurements were made in this study.

Using LC–MS/MS requires homogenisation of the tissue hence results in a loss of anatomical and spatial information but allows the analysis of a larger sample and thereby can provide increased sensitivity. Conversely, MALDI-MS imaging provides spatial and regional information but is limited by the small sampling size. Due to the small sampling size used (typically 100 µm × 100 µm) achieving sufficient sensitivity in the clinical study proved difficult and LC–MS/MS analysis was used to confirm drug was present in biopsies. Although current MALDI-MS imaging sensitivity was generally unable to fully profile drug distribution in the TBCs, it was sufficiently sensitive to detect ipratropium in certain foci. The requirement for the coincident presence of both fragment ions in the MALDI-MS imaging data, at a signal to noise ratio threshold of 3:1 or greater as the threshold for the identification of ipratropium to be positively recorded as well as the fact that ipratropium was detected by LC–MS/MS in the remaining biopsy fraction for all distal lung biopsy samples, provides increased confidence that ipratropium was detected by MALDI-MS imaging. LC/MS was able to detect ipratropium in all distal lung biopsy samples. The sensitivity of MALDI MS is such that demonstrating colocalization with areas of histological fibrosis is more challenging. We were delighted to show this overlap in 75% of the fibrotic samples.

MALDI-MS imaging detected ipratropium in four participants’ TBC samples (Fig. 3), three of whom also had fibrotic regions identified within the TBC research samples. In some instances, e.g., Figure 4D (iii), the foci of ipratropium are not directly overlying the biopsy sample. This is likely due to diffusion/delocalisation of ipratropium from the periphery of the sample section during the sample freezing process within the embedding material and/or during the thaw-mounting process of the sample section onto the glass slide in preparation for MALDI-MS imaging. It is the authors’ opinion that this still constitutes the positive identification/detection of ipratropium in the sample section.

In all five participants, MALDI-MS imaging detected ipratropium in the endobronchial samples. More ipratropium foci and higher ipratropium signal intensities were detected in the proximal lung samples than distal lung samples even though proximal lung samples were smaller in size. This was expected, as in general inhaled drugs emitted from a device generating polydisperse particle sizes are more likely to deposit higher amounts of drug in the proximal lung and larger airways than the distal lung and alveoli [17]. In addition, with just a single dose of nebulised drug and only 10–30% of nominal dose expected to reach the lung (due to the efficiency of the nebuliser device) [20] and the estimated surface area of the human lung varying between 50 and 75 m^2^ [21], it is expected to be challenging to detect drug deposited in 5 mm^2^ distal lung TBC samples and if detected, would likely be close to the limit of the detection of any MALDI-MS imaging technique.

The deposition of an inhaled drug depends on the particle size distribution, inhaler device used and patient performance. In general, the nature of ILD may favour an inhaled drug approach. In fibrotic ILD the airways may be of wider calibre than normal due to airway splinting and distal traction bronchiectasis. In addition, FEV1 is preserved, and patients are usually able to generate reasonable inspiratory pressures required to use an inhaler. We observed minimal endobronchial secretions was at bronchoscopy to interfere with drug deposition which contrasts to the situation in airways diseases, such as asthma, that may be complicated by mucus plugging.

Our study was performed using a monodisperse inhaler and other studies using aerosolised drugs have shown that smaller particles achieved greater total lung deposition (1.5 µm [56%], 3 µm [50%], and 6 µm [46%]), farther distal airways penetration (0.79, 0.60, and 0.36, respective penetration index), and more peripheral lung deposition (25, 17, and 10%, respectively) [22]**.** As well as nebulisers the other main types of inhaler devices are metered-dose inhalers (MDIs) and dry drug powder inhalers. Current inhalers generally have a broad particle distribution (0.5–6 µm), comparable to the nebuliser. The Respimat is a reusable soft mist MDI with a higher fine particle fraction (about 2.5-fold) and a slower velocity (× fivefold) compared to propellant-driven MDIs. It delivers approximately 60–70% of its dose in the respirable particle fraction (< 1.0 µm) and is the only commercial device to deliver particles < 0.3 µm. It is therefore likely that a soft mist MDI might allow delivery of drug even deeper into the lung, but this was beyond the scope of this study.

This proof of concept study studied a small number of patients, balancing risk of research biopsies against benefits of understanding inhaled drug distribution in fILD, and has several limitations. There was a difference between the demonstrated detection of ipratropium in the pre-clinical study versus the clinical study, despite using what was considered a scaled dose. The human ipratropium dose of 500 mcg was converted to 0.5 mcg/g in lung tissue by assuming a human lung weight of 1000 g. A similar assumption was made for rat lung weight of 1.5 g and the 0.5 mcg/g lung tissue dose was matched between the species. As this was an experimental study, we were not in a position to quantify the rat to human “disconnect”; we do not have systemic (plasma) data or quantified human lung concentrations. Indeed, the pre-clinical work was only performed to allow study sample workup and methodologies to be put in place. Possible explanations for the observed “disconnect” could be the effect of impaired lung function of the participants, pulmonary clearance mechanisms, or a degree of wash out of drug due to the administration of topical anaesthetic during the bronchoscopy. In the pre-clinical rat study, the lung levels for ipratropium appeared to be consistent throughout the 5–65 min time period. We assumed that this would be the same in humans, but this may not be the case. The delay of up to 60–70 min before biopsy may have contributed to some dissolution and absorption of the ipratropium in the airways. However, whilst topically active, ipratropium as a quaternary ammonium compound, is poorly absorbed [23] but has a reported short systemic half-life of 1.6 h [24]**.** Exact correlation with the underlying histopathology was sometimes confounded due to delocalization of drug, presumably during sample processing, together with limitations to the histological assessments resulting from the use of the embedding material and section thickness needed for sample preparation. While we were able to prove that inhaled ipratropium does deposit in distal, fibrosed lung in participants with ILD, we were not always able to show the exact location within the biopsy samples with confidence. As we were operating close to the limits of detection of the current instrument (MALDI), we could not show the potential drug distribution. Therefore, in further studies we would recommend use of an increase in drug dose and/or greater MALDI-MS sensitivity.

The advent of TBC has brought translational research opportunities by allowing minimally invasive and rapid access to lung interstitial tissue and therefore the potential to study relatively large distal lung biopsies without the need for a Video Assisted Thoracoscopic Surgery or open surgical approach. A further advantage over surgical acquisition of samples is the fact that participants are self-ventilating throughout the procedure which in this study should lead to a more physiological drug distribution than in ventilated participants. Time from nebulisation to biopsy is also reduced as the participant can be nebulised in the bronchoscopy suite directly before receiving sedation.

In this proof of concept study, we are able to present confirmation that inhaled drug therapy is a feasible route of administration for fibrotic ILD. However, further work is needed to encompass the influences of the varying physicochemical properties of different pharmaceutical formulations to be used in IPF to optimise distal delivery. Similarly, development of an inhaled therapy would also require an understanding and evaluation of drug clearance particularly since fibrotic interstitium between the alveolar epithelium and the blood supply would likely impair drug penetration into the blood vessels.

Future studies using this unique and the powerful combination of TBC and Mass Spectrometry have the potential to evaluate the ability of an inhaled, or systemic dosed molecule to reach the lung, and may in particular shorten the early clinical phase of an inhaled drug where target engagement is important to demonstrate early in development.

## Conclusion

We have demonstrated in this study for the first-time using LC–MS/MS and MALDI-MS imaging that a drug taken via the inhaled route can deposit in distal fibrotic lung tissues. All participants had a fibrotic ILD with overall moderately impaired lung function. To our knowledge, this is the first study to directly assess the deposition of non-radiolabeled drugs to the distal lungs of participants with ILDs and correlating histology with drug deposition in these participants.

Ipratropium was detected in all TBC and endobronchial samples tested indicating that drug deposition reached the peripheral lung, a region that is most affected in IPF.

This study, therefore, in addition to the study by Usmani et al., 2018 [17] suggests that ILD participants with established fibrosis can benefit from treatments administered by the inhaled route.

## Take home message

Using mass spectrometry, this study demonstrates for the first-time that an inhaled drug can deposit in distal fibrotic lung parenchyma in patients. This finding suggests that drugs to treat pulmonary fibrosis could potentially be administered by the inhaled route.

## Supplementary Information

**Additional file 1.**Online data supplement.

## Data Availability

The datasets used and/or analysed during the current study are available from the corresponding author on reasonable request. Within 6 months of this publication, anonymized individual participant data, the annotated case report form, protocol, reporting and analysis plan, data set specifications, raw dataset, analysis-ready dataset and clinical study report will be available for research proposals approved by an independent review committee. Proposals should be submitted to www.clinicalstudydatarequest.com. A data access agreement will be required.

## Abbreviations

AEs: Adverse events
F: Fibrotic
H&E: Haematoxylin and eosin
ILD: Interstitial lung disease
IPD: Idiopathic pulmonary fibrosis
LC–MS/MS: Liquid chromatography—tandem mass spectrometry
MALDI-MS: Matrix assisted laser desorption ionisation—mass spectrometry
TBC: Transbronchial cryobiopsy
