# Supplementary material for: Mass spectrometry detection of inhaled drug in distal fibrotic lung
Source: Respir Res. 2022 May 11;23:118. doi: 10.1186/s12931-022-02026-5 (PMC9092847; doi:10.1186/s12931-022-02026-5)

**Online Data Supplement**

**Mass Spectrometry detection of inhaled drug in proximal and distal regions of fibrosed human lung.**

**Mikolasch Theresia A^1&7^, Oballa Eunice^2^, Vahdati-Bolouri Mitra^3^, ~~Morrell Josie~~^~~4~~^, Jarvis Emily^4^, Cui Yi^5^, Cahn Anthony^2^, Terry Rebecca L^6^, Sahota Jagdeep^1&7^, Thakrar Ricky^7^, Marshall Peter ^8*^, Porter Joanna C^1&7*^**

**Introduction**

The MALDI-MS imaging experiments involve the ionization of molecules in a raster from sections of biopsies placed on conductive glass slides using a pulsed laser. The resulting gas-phase analyte ions are separated according to their mass to charge ratio (*m/z*) by a mass analyser. The addition of an organic matrix to the surface of the sample section is used to increase ionisation efficiency and for the extraction of analytes from the tissue sample. The focus of MALDI-MS imaging is to obtain chemical information (in this instance the detection of ipratropium) from a tissue section with the highest spatial resolution and at biological relevant sensitivities, without compromising the original spatial distributions of the drug(s)/components of interest. The spatial resolution achieved by MALDI-MS imaging is limited by the size of the matrix crystal deposited on the sample section and the laser spot size of the instrument.

Ipratropium bromide was selected for this study as it readily ionises, the limits of detection (LOD) for ipratropium in the MALDI-MSI experiments was determined as 1 pg/µL spotted onto a rat lung tissue section. For LC-MS/MS, the lower limit of quantification (LLOQ) measured for ipratropium in the non-clinical study was 2 pg/rat lung section and 1 pg/5 biopsy sections. Also, the MS/MS fragmentation pattern for ipratropium produces two fragment ions (at m/z 166.0 and 123.9, see **Online Data Supplement Figure E1**).

After MALDI-MS imaging analysis was complete, a histopathological assessment of the biopsy section (representing the lung parenchyma) was made, to evaluate the degree of fibrosis and inflammation in the biopsy and therefore the lung. This also allowed the correlation of specific ion images (i.e. drug distribution profiles) with the histological features observed by optical microscopy and digital scanning instruments on the same section. To achieve the histopathological assessment, the MALDI matrix was removed from the sample using ethanol washes and the H&E staining procedure performed, if sample integrity was maintained. Note: this procedure can result in poor histology and thus it may be useful to compare to the adjacent histology section.

**Methods**

**Chemicals**

HPLC grade xylene, methanol. ethanol, water and disposable moulds were purchased from Fisher Scientific UK Ltd (Loughborough, UK). Trifluoroacetic acid was obtained from Acros Organic (Fair Lawn, NJ, USA). Poly[N-(2-hydroxypropyl)-Methacrylamide], Carboxymethylcellulose sodium salt (medium viscosity), Eosin Y solution alcoholic, hematoxylin solution Gill No.2, clarifier solution and bluing reagent were purchased from Sigma Aldrich (Gillingham, UK). The matrix compound, α-cyano-4-hydroxycinnamic acid (CHCA), was obtained from Bruker Daltonics (Bremen, Germany).

**Pre-clinical support study Wistar rats**

All animal studies were ethically reviewed and carried out in accordance with UK Animals (Scientific Procedures) Act 1986, European Directive 2010/63/EU and the GSK Policy on the Care, Welfare and Treatment of Laboratory Animals.

Animal exposure – a 0.045 mg/ml (parent) ipratropium solution in saline (dose achieved = 1.15 µg) was administered as a nebulised solution (Pari nebuliser, air flow 7 L/min). Animals were euthanised; immediately after inhalation, 15 min, 35 min and 65 min, n=2 rats per time point. Lungs were removed with the trachea intact and 5 mm biopsy punch tissue samples were taken (**Online Data Supplement Figure E2**) and embedded into Poly[N-(2-hydroxypropyl)-methacrylamide] (pHPMA) embedding polymer, frozen and stored at -80˚C prior to MALDI-MS imaging and histology.

The frozen blocks of embedding polymer were mounted onto the chuck of a Leica 3050S Cryostat (Leica Microsystems, Wetzlar, Germany) using carboxymethyl-cellulose (CMC) (1 % w/v aqueous) ensuring the tissue region of interest was not embedded within the CMC. Consecutive sections of 16 µm thickness were cut and thaw-mounted continuously in the following order: a section mounted onto an indium tin oxide coated glass slide (Bruker Daltonics, Bremen, Germany) for MALDI-MS Imaging and the next section thaw-mounted onto a frosted end microslide (Menzel-Glaser Superfrost®, Thermo Fisher Scientific, Waltham, MA, USA) for Histology, H&E staining. The glass mounted tissue sections were optically scanned in a Super CoolScan 5000ED scanner fitted with a MA-21 slide mount adapter (Nikon Corporation, Tokyo, Japan) to produce a digital image for future reference.

**Clinical study samples**

We conducted a prospective clinical study approved by London Camden and Kings Cross Research Ethics Committee and registered on clinicaltrials.gov (NCT03136120). This study was conducted at University College London Hospital (UCLH), London, United Kingdom and sponsored by GlaxoSmithKline. Seven participants were enrolled between November 2017 and November 2018. Participants over the age of 18 with suspected ILD and requiring TBC for further diagnostic assessment, as determined by the ILD multidisciplinary team, were eligible to participate.

Inclusion criteria:

- 18 and above years of age inclusive, at the time of signing the informed consent.
- Participants with suspected ILD listed for TBC for clinical reasons following review by the ILD services at University College London Hospitals in whom diagnosis has remained unclear following radiological and clinical assessment.
- Capable of giving signed informed consent which includes compliance with the requirements and restrictions listed in the consent form and in this protocol.
- Male or Female.

Exclusion criteria:

- Participants who have a known drug allergy or other contra-indication to Ipratropium bromide
- Known hypersensitivity to atropine or Ipratropium bromide or any other known drug allergies that, in the opinion of the investigator or GSK Medical Monitor, contraindicates their participation.
- As a result of the medical history, physical examination or screening investigations, the physician responsible considers the participant unfit for the study.
- The participant is unable or unwilling to perform study assessments and procedures correctly.
- Participants with a recognised co-existing respiratory disorder (other than ILD) that in the opinion of the investigator would confound the study outcomes.

All participants received a single dose of 500 mcg nebulised ipratropium bromide via Phillips Porta Neb device (Amsterdam, Netherlands) with a SideStream aerosolising chamber (Respironics, Tangmere, UK) within approximately one hour of the start of bronchoscopy. As recommended by the European Respiratory Society guidelines on the use of nebulisers (**E1**), the nebuliser was run until the low volume in the nebulising chamber caused spluttering or 10 minutes of sputtering was completed, whichever occurred earlier. The typical volume left was 0.5 mL to 1.0 mL. It is not recommended to run a nebuliser to dryness.

TBCs for diagnosis were performed as per standard local procedure using an ERBECRYO 1 unit with a 2.4 mm cryoprobe (Tübingen, Germany). Samples were taken in radiologically pre-selected fibrotic areas by passing the cryoprobe through a terminal bronchus to reach lung parenchyma approximately 1 cm from the pleural edge. Next, one to two additional TBC research samples were taken. TBC are parenchyma biopsies and therefore expected to contain alveolar tissue as well as bronchioles, alveolar ducts and vasculature. Up to three endobronchial forceps biopsy samples were taken at the level of the right secondary carina as positive controls and to allow a comparison of proximal and distal drug deposition. Endobronchial biopsies contain airway wall comprising of epithelium, underlying basement membrane with associated vasculature and smooth muscles. The bronchoscopist determined the exact number of biopsies taken according to participant safety and the quality of the obtained specimens. Each study sample was individually embedded in cold (~4 °C) Poly[N-(2-hydroxypropyl)-methacrylamide] (pHPMA) immediately after collection [**E2**], frozen on dry ice and stored at -80˚C.

As per standard clinical practice participants were monitored for safety continuously during the procedure and had a chest X-Ray and clinical review approximately 2 hours after the biopsy to exclude a pneumothorax. They had a telephone consultation 7 days post procedure to assess for late adverse events.

**Biopsy Sample Processing**

Frozen TBC and endobronchial forceps biopsy samples taken for research were removed, as quickly as possible to minimise thawing, from the cryobiopsy probe and immediately embedded individually into the Poly[N-(2-hydroxypropyl)-methacrylamide] embedding polymer as described in Strohalm et al, 2011 [**E2**] and frozen as detailed below. 10mL of 15% (w/v) Poly[N-(2-hydroxypropyl)-methacrylamide] (pHPMA) was prepared in HPLC grade water and stored at 4°C for at least an hour before use. Prepared pHPMA can be stored at 4°C for up to 3 months.

0.7 mL pHPMA solution was dispensed to the centre of a disposable mould and the cryobiopsy sample immersed into the solution and immediately placed on dry ice to freeze. Once the solution containing the biopsy sample is frozen, the mould and contents were stored in a suitable, labelled, container/plastic bag in a -80˚C freezer.

There were several instances where the foci of ipratropium were not directly overlying the biopsy sample, particularly evident in the endobronchial samples. (Figure 3). In MALDI-MS imaging experiments it is essential to maintain the original spatial distribution of the drug and thus any diffuse/delocalisation is undesirable. This off-tissue effect is likely caused because the pHPMA is a liquid at 4 °C and the thermal mass of the 0.7mL volume may have been sufficient to thaw the extremities of the TBCs which at the point of sampling were at between 80 – 89 °C on the cryoprobe. The endobronchial forceps biopsy samples, however, were not frozen at the point of sampling and were smaller in size than the TBCs and thus would be more prone to thawing, resulting in a greater likelihood of drug diffusion/delocalization occurring during the freezing of the block than observed for the TBCs.

**Biopsy Sectioning**

The frozen block of embedding material containing the biopsy was removed from the mould and mounted onto the chuck of a Leica 3050S Cryostat (Leica Microsystems, Wetzlar, Germany) using carboxymethylcellulose (CMC) (1 % w/v aqueous) ensuring the block was not immersed within the CMC. Consecutive sections of 10 µm thickness were cut and thaw-mounted continuously in the following order: a section mounted onto an indium tin oxide coated glass slide (Bruker Daltonics, Bremen, Germany) for MALDI-MS Imaging and the next section thaw-mounted onto a frosted end microslide (Menzel-Glaser Superfrost®, Thermo Fisher Scientific, Waltham, MA, USA) for Histology, H&E staining. The glass mounted tissue sections were optically scanned in a Super CoolScan 5000ED scanner fitted with a MA-21 slide mount adapter (Nikon Corporation, Tokyo, Japan) to produce a digital image for future reference.

**Histopathology**

The biopsy sections were stained with haematoxylin and eosin (H&E) following standard histological procedures derived from Lillie *et al* [**E3**]. The procedure used is outlined below:

1. If a section was not used for MALDI-MS proceed directly to step 2 below. For sections used for MALDI-MS, first wash the section twice using 70% ethanol and then 100% ethanol. This step to removes the matrix that was applied to the slide prior to performing MALDI-MS imaging.
2. If necessary, allow the slides to equilibrate to room temperature
3. Immerse slides for 1 minute in 10% neutral buffered formalin
4. Then, 30 seconds in hematoxylin-2
5. Rinse for 1 minute with running tap water
6. Immerse slides for 10 seconds in clarifier solution
7. Rinse for 1 minute with running tap water
8. Immerse for 1 minute in bluing reagent
9. Immerse for 15 seconds in Eosin-Y
10. Rinse for 1 minute with running tap water
11. Dehydrate with ethanol
12. Dehydrate with Xylene
13. Slides should be covered with a coverslip prior to scanning using a suitable glue (e.g. Cytoseal).

**Liquid Chromatography – Tandem Mass Spectrometry (LC-MS/MS) analysis**

**Pre-Clinical Support in Wistar Rats**

During MALDI-MS imaging section preparation, sets of 5 sections were collected together in a 2mL Precellys homogenising tube that was supplied prefilled with CK28 ceramic beads. 0.5 mL of water was added to each tube before it was homogenised using a Precellys homogeniser (2 x 10 seconds, 5000 rpm). The samples were extracted by protein precipitation and the extracts were analysed by LC-MS/MS (Shimadzu Nexera UPLC, Sciex API6500+; Acentis Express 2.7 µm C18 2.1 x 50 mm UPLC column).

Sets of 5 sections of control lung were collected together in a 2mL Precellys homogenising tube that was prefilled with CK28 ceramic beads. These were used to prepare control lung homogenate using the same homogenisation and extraction method as for samples.

A 10 ng/5 sections top calibration standard was prepared by adding 10 µL of a 1 µg/mL solution of ipratropium bromide in 10/90 (v/v) acetonitrile / water directly onto a set of 5 sections of control lung in a 2mL Precellys tube supplied prefilled with CK28 ceramic beads. 0.5 mL of water was added to the tube before it was homogenised using a Precellys homogeniser (2 x 10 seconds, 5000 rpm). A set of calibration standards covering the range 0.001 to 5 ng/5 sections was prepared by further serial dilution of the 10 ng/5 section top calibration standard with control lung homogenate. Calibration standards were extracted by protein precipitation and the extracts were analysed by LC-MS/MS (Shimadzu Nexera UPLC, Sciex API6500+; Acentis Express 2.7 µm C18 2.1 x 50 mm UPLC column).

**Clinical Study**

Each biopsy sample was individually thawed, removed from the embedding material and weighed, then homogenised in a fixed volume of water (irrespective of the sample weight) at 5000 rpm in 2 mL Precellys tubes containing ceramic beads (Precellys 24, Berin Instrument). The samples were extracted by protein precipitation and the extracts were analysed by LC-MS/MS (Shimadzu Nexera UPLC, Sciex API6500+; Kinetex EVO 2.6 µm C18 2.1 x 50 mm column).

Ipratropium was identified by LC-MS/MS using the LC retention time of the drug and the predetermined specific mass transitions for ipratropium.

**MALDI MSI analysis**

Tissue sections were coated with ~1-2 mL of 7 mg/mL CHCA matrix solution in 70 % MeOH/0.2 % TFA (aq.) (Imageprep, Bruker Daltonics). LIFT MS/MS spectra were acquired in positive ionisation mode using a Smartbeam II laser, 200 laser shots per position (RapifleX or UltrafleXtreme MALDI TOF/TOF, Bruker). Mass spectrometer parameters were as per the manufacturers recommended settings, adjusted for optimal performance. Data analysis was carried out using FlexImaging v 4.0. Predetermined specific mass transitions for ipratropium (m/z 332.2-166.0 and 332.2-123.9) were utilised. Following smoothing and baseline correction, a signal to noise threshold ratio of 3:1 was applied to both fragment ions (166.0 and 123.9) for detection of ipratropium. This detection criterion was based upon the analysis of the control rat lung (Rat #10 - undosed) from the pre-clinical study and provided a sufficient cut-off to eliminate the occurrence of any false positives.

**Results**

Ipratropium was measured in rat lung sample sections by LC-MS/MS with the mean amount across samples from all timepoints up to 65 minutes post administration in the region of 100 pg/lung section and <10 pg/biopsy section.

Positive mode ionisation was used for the MALDI-MS imaging experiments and the analysis reported herein is for the ipratropium cation (and will be referred to as ipratropium or drug). Ipratropium was identified in the biopsy sections by MS/MS using predetermined specific mass transitions for ipratropium (i.e. m/z 332.2-166.0 and 332.2-123.9).

Following the pre-clinical study, we elected to take endobronchial biopsy samples, to use as a positive control. The levels of ipratropium were expected to be higher in the proximal lung than in the distal lung, thus if we were unable to detect ipratropium in the proximal lung it would be unlikely to be detected in the distal lung.

MALDI-MS Imaging experiments were performed using a spatial resolution of either 30 µm, 100 µm or 200 µm and the signal of the analyte was displayed using a colour coded ion density map.

**References**

E1. Boe J, Dennis JH, O’Driscoll BR, Bauer TT, Carone M, Dautzenberg B, Diot P, Heslop K, Lannefors L. European Respiratory Society guidelines on the use of nebulisers. European Respiratory Journal 2001; 18(1): 228-242.

E2. Strohalm M, et al. Poly[N-(2-hydroxypropyl)methacrylamide]-Based Tissue-Embedding Medium Compatible with MALDI Mass Spectrometry Imaging Experiments. Anal. Chem. 2011; 83: 5458-5462.

E3. Lillie RD, Pizzolato P, Donaldson PT. Nuclear stains with soluble metachrome metal mordant lakes. Histochemistry 1976; 49: 23-35.

**Supplement Figure Legends**

**Supplement Figure E1** – Ipratropium (structure and fragmentation).

**Supplement Figure E2** – Pre-Clinical Study in Rat - Rat Lung taken immediately after ipratropium dosing (Rat 1), with multiple 5 mm punched biopsies removed.

**Figures**

**Supplement Figure E1** – Ipratropium (structure and fragmentation)


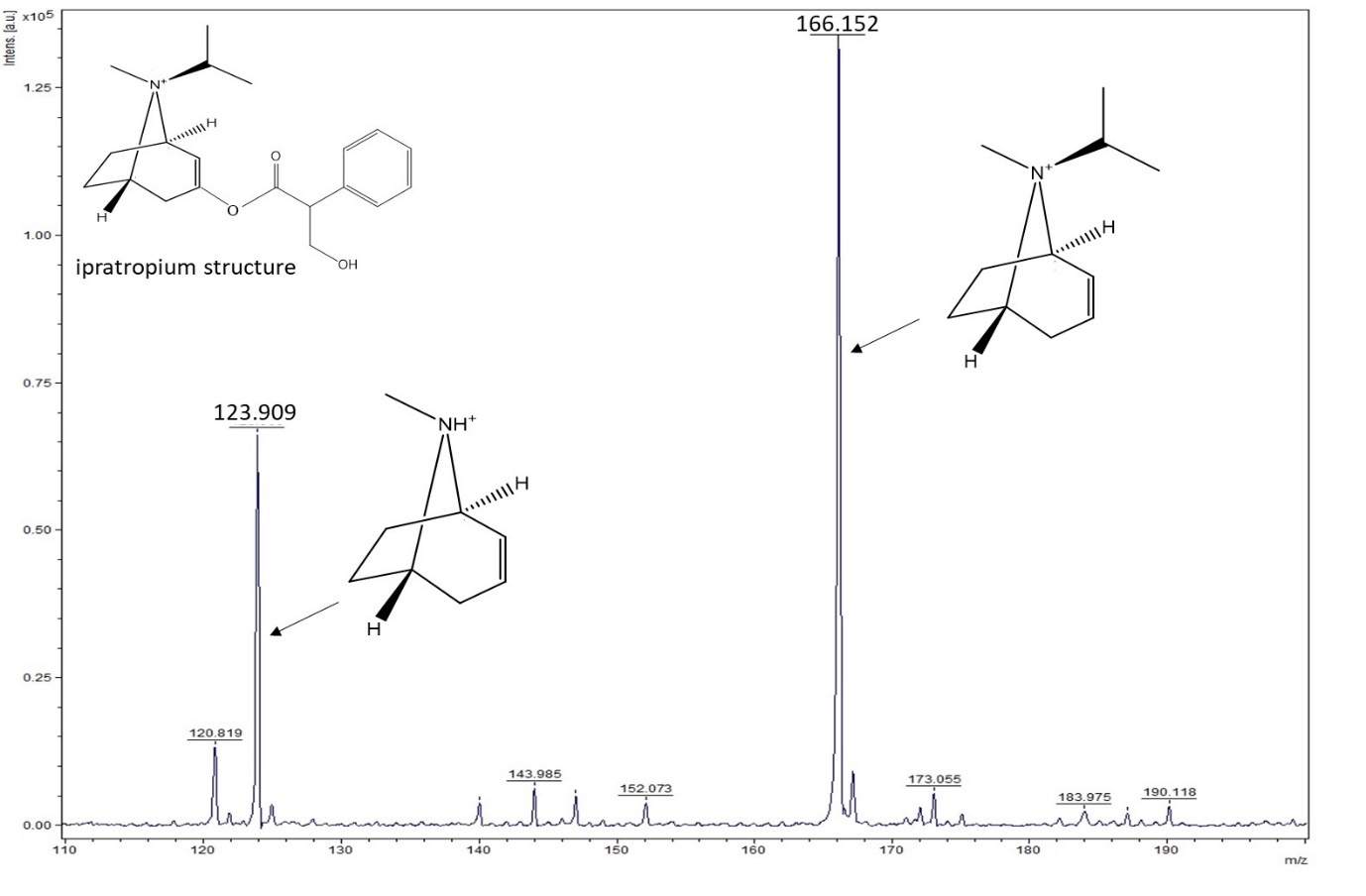


**Supplement Figure E2**


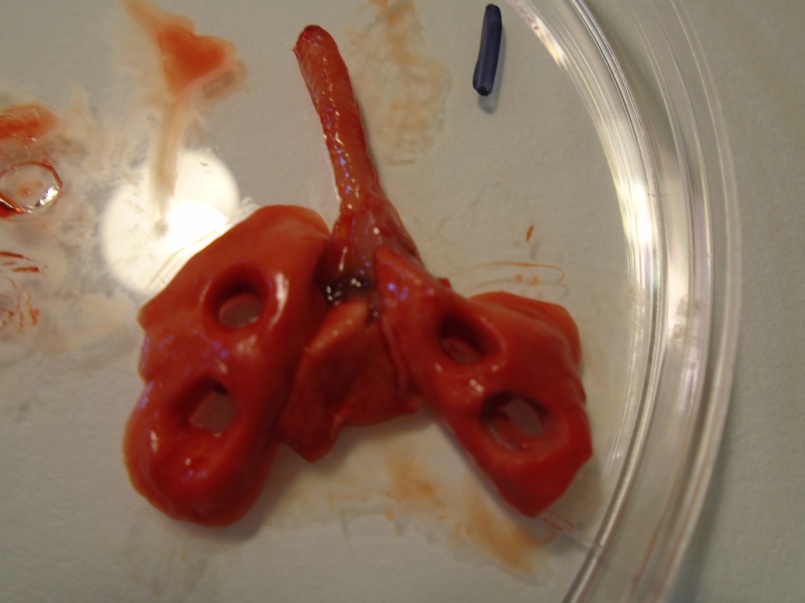

Supplement: Supplementary file 1 — Additional file 1.Online data supplement. [file 12931_2022_2026_MOESM1_ESM.docx]
